# H_2_O_2_-Elicitation of Black Carrot Hairy Roots Induces a Controlled Oxidative Burst Leading to Increased Anthocyanin Production

**DOI:** 10.3390/plants10122753

**Published:** 2021-12-14

**Authors:** Gregorio Barba-Espín, Christian Martínez-Jiménez, Alberto Izquierdo-Martínez, José R. Acosta-Motos, José A. Hernández, Pedro Díaz-Vivancos

**Affiliations:** Department of Fruit Breeding, CEBAS-CSIC, Campus Universitario de Espinardo, 30100 Murcia, Spain; christianjose.martinezj@um.es (C.M.-J.); albertoizquierdo998@gmail.com (A.I.-M.); jacosta@cebas.csic.es (J.R.A.-M.); jahernan@cebas.csic.es (J.A.H.); pdv@cebas.csic.es (P.D.-V.)

**Keywords:** antioxidant metabolism, elicitation, hydrogen peroxide, in vitro culture, superoxide dismutase, reactive oxygen species (ROS) scavenging

## Abstract

Hairy roots (HRs) grown in vitro are a powerful platform for plant biotechnological advances and for the bio-based production of metabolites of interest. In this work, black carrot HRs able to accumulate anthocyanin as major secondary metabolite were used. Biomass and anthocyanin accumulation were improved by modulating growth medium composition—different Murashige & Skoog (MS)-based media—and H_2_O_2_-elicitation, and the level of the main antioxidant enzymes on elicited HRs was measured. Higher growth was obtained on liquid 1/2 MS medium supplemented with 60 g/L sucrose for HRs grown over 20 days. In this medium, 200 µM H_2_O_2_ applied on day 12 induced anthocyanin accumulation by 20%. The activity of superoxide dismutase (SOD)—which generates H_2_O_2_ from O_2_^•−^—increased by over 50%, whereas the activity of H_2_O_2_-scavenging enzymes was not enhanced. Elicitation in the HRs can result in a controlled oxidative burst, in which SOD activity increased H_2_O_2_ levels, whereas anthocyanins, as effective reactive oxygen species scavengers, could be induced to modulate the oxidative burst generated. Moreover, given the proven stability of the HR lines used and their remarkable productivity, this system appears as suitable for elucidating the interplay between antioxidant and secondary metabolism.

## 1. Introduction

Infection with the soil-borne pathogenic bacterium *Rhizobium rhizogenes* (formerly *Agrobacterium rhizogenes*) causes the proliferation of “hairy roots” (HRs) on a range of dicotyledonous plants [1,2]. Upon infection, T-DNA from the so-called root-inducing (Ri) plasmid, is inserted and integrated in the host plant DNA [1]. The transfer and expression of four loci from the Ri plasmid, commonly referred as *rol*-genes, play a key role in development of characteristic HRs at the infection site [3,4]. Based on the naturally occurring *R. rhizogenes* rol-genes, the initial transformation steps without the use of recombinant DNA can be termed “natural transformation”, and products derived from this platform are considered as non-genetically modified organisms [5].

The remarkable advantages of HRs cultures derived from infected plants include: (1) rapid and high-density growth in hormone-free medium, (2) potentially increased production of secondary metabolites compared to the starting plant material, (3) genotypic stability of the derived HR lines, and (4) simple separation of biomass from the nutrient medium [1,6,7,8,9]. Currently, HR-based production of secondary metabolites is a hotspot in plant biotechnology. Likewise, HRs are a useful platform for gene function studies and to investigate physiological processes, among other applications [4,10]. In consequence, over the last three decades, there has been an increasing body of research related to HR cultures [4].

Anthocyanins are water-soluble flavonoids that confer colors from red to blue to fruits, flowers, and vegetables. To date, over 600 different anthocyanins have been identified from plant sources comprising six common aglycones and various glycosylated and acylated compounds [11,12]. There is increasing demand for natural food colorants that can substitute synthetic colors due to both legislative actions and consumer concerns. Highly stable acylated anthocyanins may provide desirable color and stability for commercial food products [11,13]. Anthocyanins are the major secondary metabolites of black carrot (*Daucus carota* L. ssp. *sativus* var. *atrorubens* Alef). Some commercial hybrids of black carrots have been reported to contain anthocyanin contents from 1.5 to 3.5 mg g^−1^ fresh weight [14,15,16,17]. For its high proportion of monoacylated structures with three sugar moieties, black carrot anthocyanins possess high chemical stability, which makes them adequate substitutes for synthetic colorants. Today, black carrot extracts are a common ingredient in the food industry as an alternative to the red azo dye Allura Red (E129) [13,18,19]. In addition, as potent dietary antioxidants, anthocyanins contribute in the prevention of diabetes, cancer, neuronal and diseases, among other illnesses [13]. In this context, there is room for new approaches that increase profitability of pigment production.

Anthocyanins are dual compounds in the sense that are both antioxidants and secondary metabolites [20]. Secondary metabolite accumulation in HR cultures is often triggered by elicitors [21,22,23,24,25], which may function as signaling molecules of plant stress responses. Many elicitor treatments have been linked with reactive oxygen species (ROS) over-accumulation, especially hydrogen peroxide (H_2_O_2_), leading to an oxidative stress that directly activates secondary metabolite formation [22,26,27,28]. Plant responses to H_2_O_2_ are of great interest due to the role of H_2_O_2_ as a signaling molecule, being perceived as an elicitor that, when added exogenously, may trigger an oxidative stress leading to secondary metabolites accumulation [29,30,31,32]. Plants have an antioxidant defense system composed of enzymatic and non-enzymatic components [33]. Antioxidant enzymes include catalase (CAT), peroxidase (POX), superoxide dismutase (SOD) and ascorbate-glutathione (AsA-GSH) cycle enzymes [33,34]. In this scenario, anthocyanin biosynthesis may take place as a positive response to oxidative stress due to its non-enzymatic antioxidant capacity of ROS scavenging [35]. Thus, studying the interplay among elicitation, secondary metabolism and antioxidant system may shed light to the accumulation of anthocyanins in HRs.

In a recent study, anthocyanin-producing HR cultures of black carrot were reported [25]. In the present work, using two of the most productive HR lines generated in the aforementioned research [25] (lines 43-R and NB-R), the optimization of biomass accumulation and anthocyanin production was conducted by means of (1) improved composition of growth medium, followed by (2) an H_2_O_2_-elicitation assay in which exogenous H_2_O_2_ was applied at different concentrations. An interplay between H_2_O_2_ elicitation and antioxidant metabolism was found. These findings highlight black carrot HRs as a suitable platform for the in vitro production of anthocyanins and antioxidants, as well as for the study of the physiological mechanisms involved in secondary metabolites elicitation.

## 2. Results and Discussion

The genetic and phenotypic stability of HRs is well reported [10,25,36]. The HR lines used in this study were obtained over five years ago and, during that time, they were maintained via regular sub-culturing without detectable loss of vigour and coloration. This confirms that stable HRs of black carrots have been developed.

**Figure 1:**
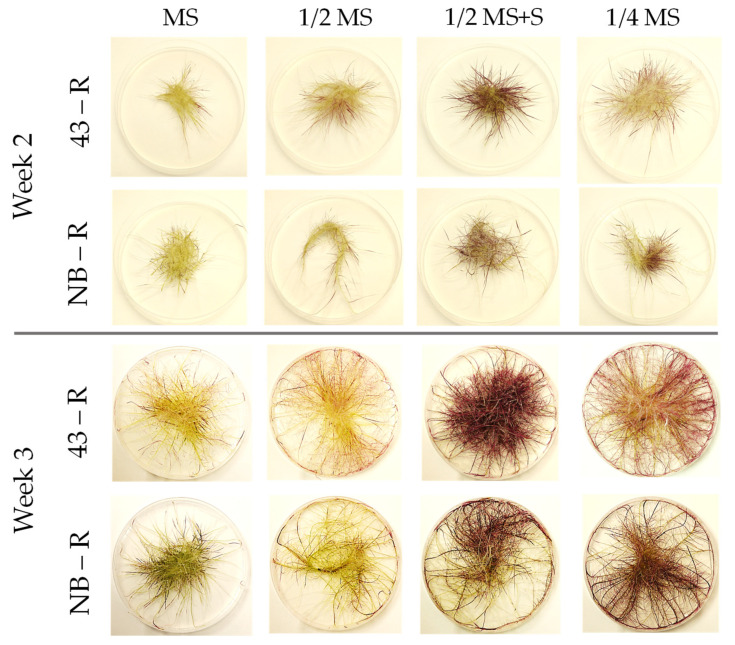
Overall appearance of black carrot hairy roots grown on solid Murashige & Skoog (MS)-based media after 2 and 3 weeks of culture. The hairy root lines NB-R and 43-R were obtained in a previous research [25].

**Figure 2:**
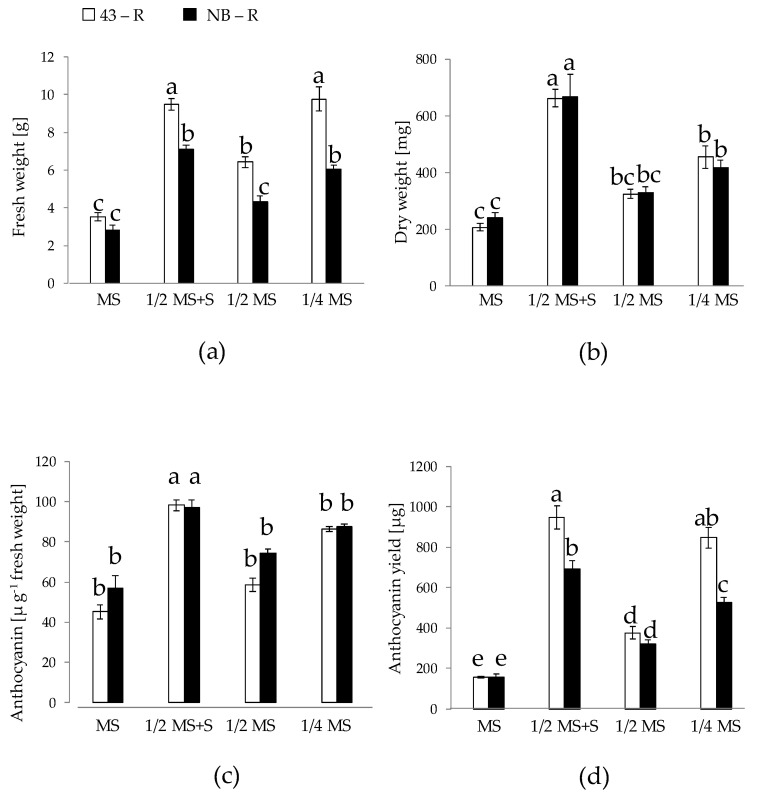
Biomass and anthocyanin production for hairy roots grown on solid Murashige & Skoog (MS)-based media over a 21-day period. The hairy root lines NB-R and 43-R were obtained in a previous research [25]. (**a**): Fresh weight; (**b**): Dry weight; (**c**): Anthocyanin concentration; (**d**): Anthocyanin yield. Data are presented as the mean ± SE of six replicates. Different letters indicate statistical differences according to Tukey’s test (*p* ≤ 0.05).

Firstly, the growth of HR lines 43-R and NB-R was compared on solid MS-based medium at different strength and composition: 1/4 MS, 1/2 MS, 1/2 MS containing 60 g/L sucrose (MS+S), and full MS. The visual aspect of the plates after 1 and 3 weeks is shown in Figure 1. Overall, higher biomass and pigmentation can be observed for the 43-R line. Moreover, 1/2 MS+S medium seemed to provide higher yields (Figure 1). These observations were confirmed by the yield measurements (Figure 2). Concerning the fresh weight (FW), the levels were higher for 43-R than for NB-R, except in MS medium where FW was equivalent (Figure 2a). In terms of dry weight (DW), no differences between lines for each medium were found (Figure 2b). Therefore, it can be deduced that differences in FW between HR lines were based on a higher water content of 43-R. This is contrary to that reported by [25] for the same HR lines, where DW yield was higher for 43-R than for NB-R, grown under a 12 h light/12 h darkness photoperiod (20 °C/18 °C). Considering the different incubation conditions in this study (16 h light, 25 °C constant), this indicates that distinct HR genotypes may respond differently to changes in environmental conditions [25,37,38]. As for the FW and DW, anthocyanin concentration was lower for HRs grown on full MS. On the other hand, anthocyanin concentration was higher for both HR lines grown on 1/2 MS+S, and for NB-R grown on 1/2 MS (Figure 2c). In this sense, an excess of nutrients could redound negatively on HR growth under our experimental conditions; this has been reported for HRs and the tissue culture of several species, where higher nutrient content in the medium either decreased or did not affected biomass accumulation [25,39,40]. Anthocyanin yield, expressed as µg anthocyanin per experimental unit (plate), can be an adequate reference to high-productive experimental conditions, as it takes into account both biomass and anthocyanin concentration. In this sense, 1/2 MS+S medium was pointed out as the one providing the highest yields, followed by 1/4 MS, 1/2 MS and full MS. Comparing HR lines, anthocyanin yield in 43-R was superior for all media tested except for 1/2 MS (Figure 2d). Again, this is consistent with the results reported previously [25] in which, under different conditions than the used in this study, 43-R provided higher anthocyanin contents than NB-R. These results led us to select 43-R line and 1/2 MS+S for elicitation experiments in liquid medium.

**Figure 3:**
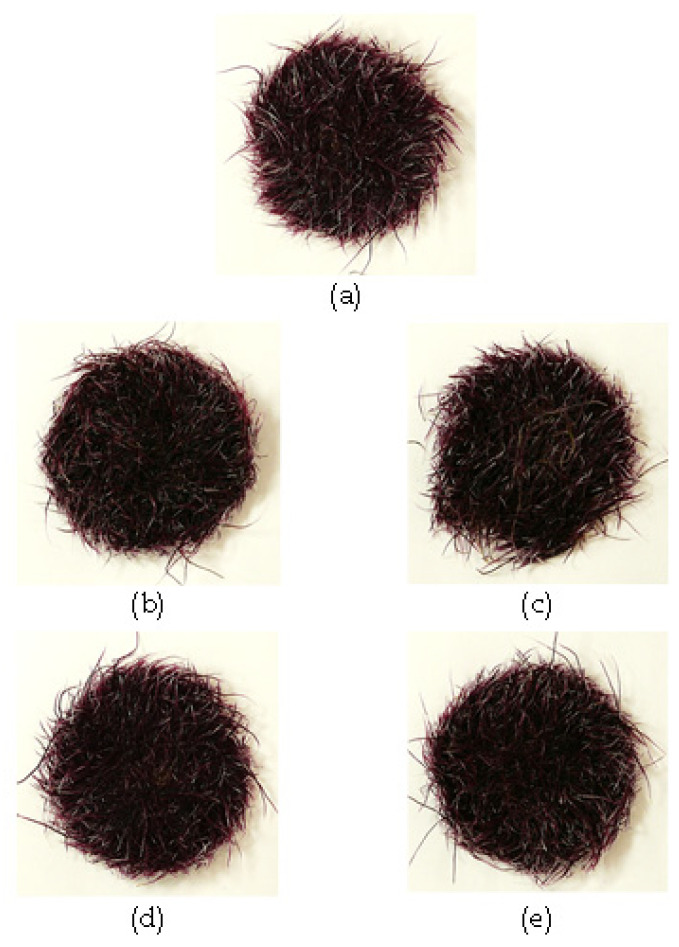
Overall appearance of black carrot hairy roots of 43-R line grown in liquid Murashige & Skoog-based medium (MS+S) over a 20-day period. (**a**) Control; (**b**): 100 µM H_2_O_2_ added on day 10; (**c**) 200 µM H_2_O_2_ added on day 10; (**d**) 100 µM H_2_O_2_ added on day 12; (**e**) 200 µM H_2_O_2_ added on day 12. The hairy root line 43-R was obtained in a previous study [25].

**Figure 4:**
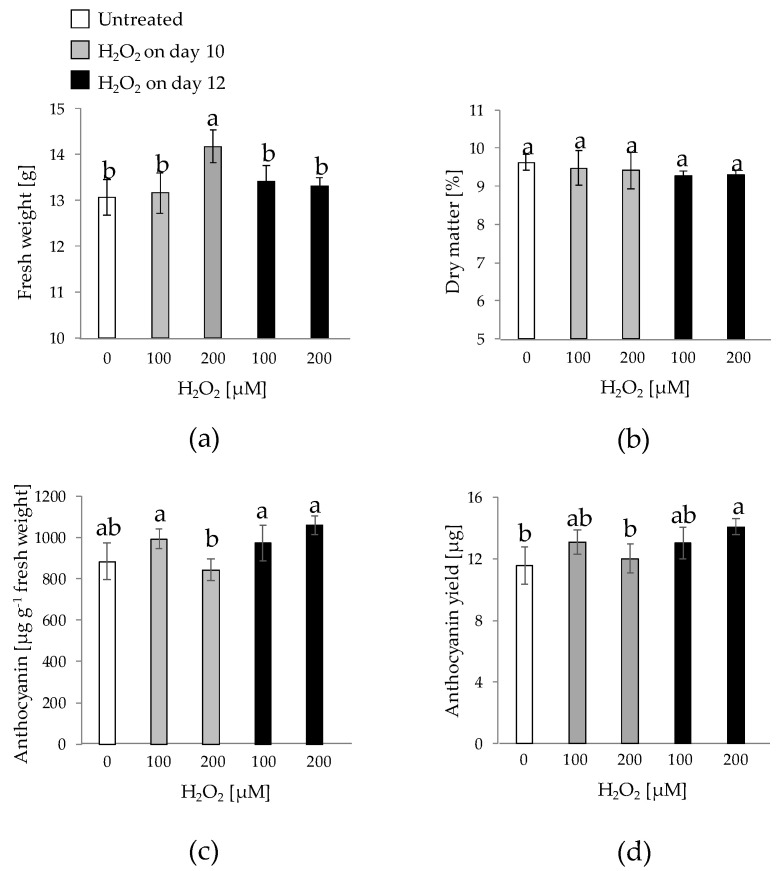
Biomass and anthocyanin production for hairy roots of 43-R grown in liquid Murashige & Skoog-based medium (MS+S) over a 20-day period with or without the addition of H_2_O_2_ (100 or 200 µM) on days 10 or 12 of culture. The hairy root line 43-R was obtained in a previous research [25]. (**a**): Fresh weight; (**b**): Dry weight; (**c**): Anthocyanin concentration; (**d**): Anthocyanin yield. Data are presented as the mean ± SE of six replicates. Different letters indicate statistical differences according to Tukey’s test (*p* ≤ 0.05).

H_2_O_2_ may accumulate transiently and generate intracellular gradients, which make it a suitable molecule for signaling roles [41]. In this study, the effect of H_2_O_2_ at two concentrations (100 or 200 µM) and two application times (days 10 and 12 of culture) was tested (Figure 3 and Figure 4). Higher H_2_O_2_ concentrations (in the mM range) were discarded, since they were deleterious for HR growth (data not shown). Visually, neither the coloration nor the size of HRs showed evident variation among the different treatments (Figure 3), although a clearly more intense coloration was perceived with respect to HRs grown on plates (Figure 2). The FW was superior for HRs treated with 200 µM H_2_O_2_ on day 10 (Figure 4a), whereas the DW was equivalent for all treatments (Figure 4b). In terms of anthocyanin concentration, a different behavior was observed: HRs treated with 200 µM H_2_O_2_ on day 10 showed the lowest anthocyanin concentration among the treatments, whereas the highest concentration (1060 µg g^−1^ FW) was achieved with 200 µM H_2_O_2_ applied on day 12. The data for anthocyanin yield highlighted these differences, with 200 µM H_2_O_2_ applied on day 12 having ca. 20% higher content than control HRs. This indicates that H_2_O_2_ may have a distinct effect depending on the application time: earlier addition (day 10) stimulated biomass accumulation while decreased anthocyanin concentration, whereas the same concentration applied on day 12 did not alter biomass while increased anthocyanin content. This may be related to a better functioning of secondary metabolism over time, as reported in two-step in vitro production systems [42]. In this sense, the elicitation effect of exogenous H_2_O_2_ on the secondary metabolism has been reported in numerous systems [43,44,45]. On the other hand, culture in liquid medium provided higher anthocyanin concentrations—an order of magnitude higher—than those observed on solid media (Figure 2), as reported in [25,46]. Moreover, the anthocyanin concentrations achieved are in the range of the observed for some field- and glasshouse-grown black carrots [14,15,47], which denotes the potential of the HR platform for the in vitro production of anthocyanins. Finally, anthocyanin yield for the 200 µM H_2_O_2_ applied on day 12 treatment was significantly higher (21.6%) than that of the control.

**Figure 5:**
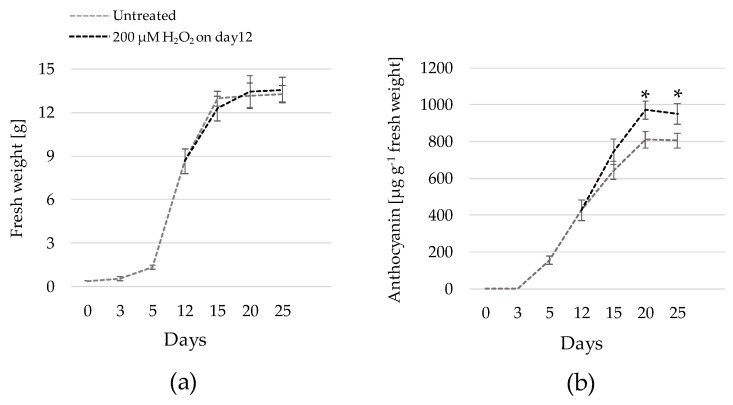
Fresh weight (**a**) and anthocyanin (**b**) accumulation curves over a 25-day period for hairy roots of 43-R grown in liquid Murashige & Skoog-based medium (MS+S) with or without the addition of 200 µM H_2_O_2_ on day 12 of culture. The hairy root line 43-R was obtained in a previous research [25]. Data are presented as the mean ± SE of six replicates. Statistical differences between sample pairs at each time point were determined according to Welch *t*-test and are denoted by an asterisk symbol.

Subsequently, in order to test the profitability of the proposed methodology, accumulation curves for both biomass and anthocyanin were performed on HRs of 43-R under control and treatment (200 µM H_2_O_2_ applied on day 12) conditions over a 25-day period (Figure 5). A growth kinetic resembling a sigmoidal curve was observed in both control and treated HRs (Figure 5a), characterized by an initial lag phase, an intermediate log phase and a plateau phase at day 20. This shape is equivalent to that previously reported on this HR line [25] and, overall, on HRs from other species [4,37,46]. Recent kinetic growth models associated HR biomass accumulation and shape of the curve with complex architecture relationships that associate the length of individual primary root with the number and length of higher-order branches [48]. Moreover, there were no significant differences on the FW between control and treated HRs at any sampling point. In contrast, anthocyanin concentration was significantly higher in treated HRs at day 20—as was also shown in Figure 4—as well as at day 25, with increases of around 20% in both cases (Figure 5b).

Taking into account that many elicitor treatments leading to enhanced secondary metabolites production have been linked to the establishment of a moderate oxidative stress [22,26,27,28], the activity of the main antioxidant enzymes was determined in 43-R line for untreated HRs, and for the two treatments that provided the highest and the lowest anthocyanin concentrations (200 µM H_2_O_2_ applied on day 12 and 200 µM H_2_O_2_ applied on day 10, respectively). The elicitation process has been linked to controlled ROS over-accumulation and oxidative burst in in vitro systems, including HRs, in which antioxidant enzymes may play a key role [22,26,27,49,50]. For instance, in broccoli cells, enhanced bioactive compound production was achieved by coronatine- and methyl jasmonate-mediated mild oxidative stress. In these elicited broccoli suspension-cultured cells, the increase in glucosinolates and phenolic compounds was correlated with higher ascorbate peroxidase (APX) activity (a H_2_O_2_-scavenging enzyme), as well as with lower monodehydroascorbate reductase (MDHAR) and glutathione reductase (GR) activities, these enzymes being part of the AsA-GSH pathway [28].

**Figure 6:**
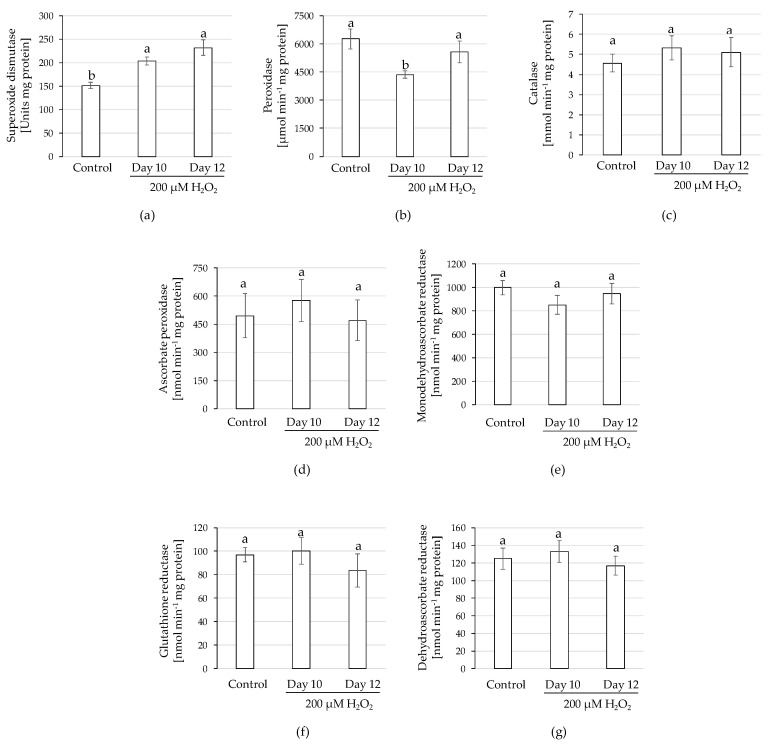
Activity of superoxide dismutase (**a**), peroxidase (**b**), catalase (**c**), ascorbate peroxidase (**d**), monodehydroascorbate reductase (**e**), glutathione reductase (**f**) and dehydroascorbate reductase (**g**) for hairy roots of 43-R grown in liquid Murashige & Skoog-based medium (MS+S) over a 20-day period with or without the addition of 200 µM H_2_O_2_ on days 10 or 12 of culture. The hairy root line 43-R was obtained in a previous research [25]. Data are presented as the mean ± SE of five replicates. Different letters indicate statistical differences according to Tukey’s test (*p* ≤ 0.05).

SOD, the first line of defence of the plant for ROS removal, catalyses the dismutation of the superoxide radical (O_2_^•−^) into O_2_ and H_2_O_2_ [51]. In this study, SOD activity was enhanced upon H_2_O_2_ elicitation in both treatments, displaying day 10 and day 12 35% and 53% increases, respectively (Figure 6a), which would favour H_2_O_2_ accumulation. This indicates a medium-term rather than a short-term effect, since increased SOD activity occurred 8 or 10 days after the H_2_O_2_ treatment. A similar behaviour was observed in methyl jasmonate-elicited cell cultures of *Pueraria mirifica* [52], where increased isoflavonoid content was associated with enhanced SOD activity (up to 56%) after three and six days of the treatment. Our results may also indicate the existence of a positive feedback loop between exogenous H_2_O_2_ and cellular H_2_O_2_ over-generation by increased SOD activity. On the other hand, the main H_2_O_2_-scavenging enzyme activities (POX and CAT) were not stimulated upon elicitation: POX activity varied among treatments; whereas H_2_O_2_ applied on day 12 did not alter POX levels with respect to the control HRs, the same treatment on day 10 significantly reduced POX activity (Figure 6b). With respect to CAT activity, no differences among treatments were found (Figure 6c). The AsA-GSH pathway play a key role in ROS elimination as well as on AsA and GSH (major non-enzymatic antioxidants in plants) recycling [33]. In this work, no significant differences were detected among the enzymes that compose the cycle: APX, MDHAR, GR and dehydroascorbate reductase (DHAR) levels were invariable under our experimental conditions (Figure 6d–f). As a result, the hypothetical H_2_O_2_ burst on treated samples would not be controlled enzymatically; instead, anthocyanins, as efficient non-enzymatic antioxidants capable of directly scavenging ROS [35] could adjust the over-generation of H_2_O_2_ in the cell (by SOD activity) or from exogenous origin (elicitation). In this sense, in Arabidopsis, ROS-induced anthocyanin production has been reported to provide feedback protection [53,54], whereas anthocyanin deficiency induced ROS-generating oxidative stress [53].

## 3. Conclusions

In the present work, the biomass and anthocyanin accumulation of black carrot hairy roots were improved by modulating growth medium composition and H_2_O_2_-elicitation. Higher biomass accumulation was achieved on liquid 1/2 MS medium supplemented with 60 g/L sucrose for HRs grown over 20 days. In this medium, 200 µM H_2_O_2_ applied on day 12 induced anthocyanin accumulation by 20%, whereas its application on day 10 decreased it. This may be related to a better functioning of secondary metabolism over time. Moreover, stimulated anthocyanin content on elicited HRs was associated with the establishment of an oxidative burst via enhanced SOD activity—a H_2_O_2_-producing enzyme—whereas the activity of other antioxidants enzymes did not change. This led us to hypothesize that anthocyanins content may be enhanced in order to cope with this oxidative burst, acting as direct ROS scavengers in elicited HRs. Given the proven stability of the HR lines used and their remarkable productivity, this system appears as suitable for the in vitro study of antioxidant and secondary metabolisms.

## 4. Materials and Methods

### 4.1. Plant Material Maintenance

The plant material used in this research, two HR lines named 43-R and NB-R, were originated from the *R. rhizogenes*-mediated transformation of black carrot cultivar ‘Night Bird’ F1 and inbred line 43, respectively, in a previous study, [25]. Therein, integration of bacterial DNA in the two lines was verified by the detection of *rolB*, as reported [25]. The HR lines were maintained via regular sub-culturing every 3 to 4 weeks on solid 1/2 Murashige & Skoog [55] (1/2 MS) medium [0.22% (*w*/*v*) MS salts including vitamins, 3% (*w*/*v*) sucrose, and 0.05% MES monohydrate] containing 7.5 g/L agar, on 9-cm diameter Petri dishes, as reported by [25], and incubated at 25 °C in the dark. For each sub-culture, younger tissue (0.4–0.45 g HR segment) was used.

As a source of inoculum for all experiments, a pre-culture was generated as follows: 0.4–0.45 g HR segments were grown in 250 mL flasks containing 100 mL of 1/2 MS for 1 week in the dark, on an oscillatory shaker (Heidolph™, Fisher Scientific, Hampton, NH, USA) at 85 rpm.

### 4.2. Cultivation on Different Solid MS-Based Media

The inoculum (0.3–0.35 g FW of a 1-week pre-culture) of both HR lines was placed onto 15 cm-plastic Petri dishes containing solid MS at different strengths [1/4 MS, 1/2 MS, 1/2 MS containing 60 g/L sucrose (MS+S), and full MS] and incubated at 16 h light (140 µmol m^−2^ s^−1^)/8 h darkness (25 °C constant) for three weeks. HRs were collected after 1 and 3 weeks of culture.

### 4.3. Growth on Liquid 1/2 MS+S Medium and H_2_O_2_ Elicitation

Based on the highest growth and anthocyanin yield obtained for R-43 on 1/2 MS+S, this line and medium were selected for further experiments on liquid medium, where a 0.3–0.35 g FW inoculum from the pre-culture was placed into 250 mL glass Erlenmeyer flasks containing 100 mL of liquid 1/2 MS+S.

First, an H_2_O_2_ stock solution was prepared and added to HR cultures in 1/2 MS+S medium at days 10 and 12 for final concentrations of 0, 100 and 200 µM. HRs were collected at day 20. Subsequently, in the light of the higher yields obtained for 200 µM H_2_O_2_ added at day 12, biomass and anthocyanin accumulations were monitored under these conditions for 25 days, collecting HRs at different times (0, 5, 10, 15, 20 and 25 days).

All flasks were incubated at 16 h light (140 µmol m^−2^ s^−1^)/8 h darkness (25 °C constant) on an oscillatory shaker (Heidolph™, Fisher Scientific) at 85 rpm.

### 4.4. HR Sampling and Processing

In all cases, the whole HR from each plate or flask (biological replicate) was collected, washed, gently dried, and weighed. Then, HRs were ground in the presence of liquid nitrogen using a mortar. The resulting powder was stored at −80 °C for further analysis.

### 4.5. Determination of Dry Matter (%) and Anthocyanin Content

A 2-g aliquot of the generated powder was blended in a 3% sulfuric acid solution (1:1, *w*/*w*). The generated homogenate was vigorously mixed with distilled water (1:2, *w*/*w*) and incubated at room temperature for 1 h. Then, the mix was centrifuged for 20 min at 4000 g. The supernatant (extract) was used to measure monomeric anthocyanin content according to the pH differential method with minor modifications [15]. Briefly, the HR supernatant was diluted in 0.2 M KCl–HCl pH 1 (1:5, *v*/*v*), and the absorption of the mix was registered between 350 nm and 700 nm using a UV/Vis V-630 Bio spectrophotometer (Jasco, Tokyo, Japan). Based on a cyanidin-3-glucoside standard, data are expressed as concentration (µg anthocyanin g^−1^ FW) and yield (µg anthocyanin per experimental unit (plate or flask)).

### 4.6. Determination of Antioxidant Enzyme Activities

Enzyme extraction was done as described [56,57,58]. Briefly, a 1-g aliquot of the HR powder was ground (1:2, *w*/*v*) into 50 mM Tris–acetate buffer containing 0.1 mM EDTA, 2 mM cysteine, and 0.2% (*v*/*v*) Triton X-100 (pH 6.0). The resulting extract was centrifuged for 15 min at 10,000× *g* (HeCttich Mikro 120, Fisher Scientific, Pittsburgh, PA, USA). Finally, the supernatant was passed through Sephadex G-25 NAP columns (GE Healthcare, Chicago, IL, USA), and the filtrate used for enzymatic determinations using a UV/Vis V-630 Bio spectrophotometer (Jasco). CAT, POX, SOD, APX, MDHAR, GR and DHAR activities were assayed according to [59,60]: CAT activity was determined by following the decrease in absorbance at 240 nm as a result of H_2_O_2_ decomposition to water and oxygen. POX activity was measured by following the oxidation of 4-metoxy-naphtol at 595 nm. SOD activity was based on the enzymatic system xanthine–xanthine oxidase, measuring the reduction of cytochrome C at 550 nm. APX activity was calculated following the oxidation of ascorbate at 290 nm. MDHAR activity was based on the reduction of ascorbate coupled to the oxidation of NADH followed at 340 nm. GR activity was measured by monitoring the oxidation of NADPH, reflected as a decrease in absorbance at 340 nm. Finally, DHAR activity was assayed by measuring the increase in absorbance at 265 nm due to the reduced ascorbate formation.

### 4.7. Statistical Analyses

Analyses were performed on five to six biological replicates. All experiments were conducted independently twice. Data were expressed as the mean ± SE. Normality and homoscedasticity of variances for all data were checked by a Shapiro and Bartlett tests, respectively. Data from single time point-experiments were compared using a one-way analysis of variance (ANOVA) followed by a Tukey HSD post hoc test (*p* ≤ 0.05), whereas data from the accumulation curves were analysed by Welch t-test. The R Program for Statistical Computing (R 3.6.3., R corp.) was used.

## Data Availability

The data presented in this study are available on request from the corresponding author.
